# A Pragmatic Non-Randomized Trial of Prehabilitation Prior to Cancer Surgery: Study Protocol and COVID-19-Related Adaptations

**DOI:** 10.3389/fonc.2021.629207

**Published:** 2021-03-10

**Authors:** Daniel Santa Mina, Daniel Sellers, Darren Au, Shabbir M. H. Alibhai, Hance Clarke, Brian H. Cuthbertson, Gail Darling, Alaa El Danab, Anand Govindarajan, Karim Ladha, Andrew G. Matthew, Stuart McCluskey, Karen A. Ng, Fayez Quereshy, Keyvan Karkouti, Ian M. Randall

**Affiliations:** ^1^ Faculty of Kinesiology and Physical Education, University of Toronto, Toronto, ON, Canada; ^2^ Faculty of Medicine, University of Toronto, Toronto, ON, Canada; ^3^ Department of Anesthesia and Pain Management, University Health Network, Toronto, ON, Canada; ^4^ Division of General Internal Medicine and Geriatrics, University Health Network, Toronto, ON, Canada; ^5^ Department of Critical Care Medicine, Sunnybrook Health Sciences Centre, Toronto, ON, Canada; ^6^ Department of Surgical Oncology, Princess Margaret Cancer Centre, Toronto, ON, Canada; ^7^ Clinical Nutrition, Princess Margaret Cancer Centre, University Health Network, Toronto, ON, Canada; ^8^ Department of Surgery, Sinai Health System, Toronto, ON, Canada; ^9^ Department of Anesthesia, St. Michael’s Hospital, Toronto, ON, Canada; ^10^ Department of Geriatrics, Sinai Health System, Toronto, ON, Canada

**Keywords:** prehabilitation, cancer, pragmatic trial, cancer surgery, health quality, implementation science, feasibility

## Abstract

**Background:**

Experimental data highlight the potential benefits and health system cost savings related to surgical prehabilitation; however, adequately powered randomized controlled trial (RCT) data remain nascent. Emerging prehabilitation services may be informed by early RCT data but can be limited in informing real-world program development. Pragmatic trials emphasize external validity and generalizability to understand and advise intervention development and implementation in clinical settings. This paper presents the methodology of a pragmatic prehabilitation trial to complement emerging phase III clinical trials and inform implementation strategies.

**Methods:**

This is a pilot pragmatic clinical trial conducted in a large academic hospital in Toronto, Ontario, Canada to assess feasibility of clinical implementation and derive estimates of effectiveness. Feasibility data include program referral rates, enrolment and attrition, intervention adherence and safety, participant satisfaction, and barriers and facilitators to programming. The study aims to receive 150 eligible referrals for adult, English-speaking, preoperative oncology patients with an identified indication for prehabilitation (*e.g*., frailty, deconditioning, malnutrition, psychological distress). Study participants undergo a baseline assessment and shared-decision making regarding the intervention setting: either facility-based prehabilitation or home-based prehabilitation. In both scenarios, participants receive an individualized exercise prescription, stress-reduction psychological support, nutrition counseling, and protein supplementation, and if appropriate, smoking cessation program referrals. Secondary objectives include estimating intervention effects at the week prior to surgery and 30 and 90 days postoperatively. Outcomes include surgical complications, postoperative length of stay, mortality, hospital readmissions, physical fitness, psychological well-being, and quality of life. Data from participants who decline the intervention but consent for research-related access to health records will serve as comparators. The COVID-19 pandemic required the introduction of a ‘virtual program’ using only telephone or internet-based communication for screening, assessments, or intervention was introduced.

**Conclusion:**

This pragmatic trial will provide evidence on the feasibility and viability of prehabilitation services delivered under usual clinical conditions. Study amendments due to the COVID-19 pandemic are presented as strategies to maintain prehabilitation research and services to potentially mitigate the consequences of extended surgery wait times.

## Introduction 

Surgery is a highly prevalent primary treatment for localized tumors. Patients undergoing cancer surgery are at risk for surgery-related morbidity and mortality. For example, the rates of mortality and significant complications within 30 days of major abdominal cancer surgery are 4 and 50%, respectively (1). Numerous health-related quality of life (HRQOL) consequences are also common after oncologic surgery and may persist for an indefinite period (2). Frail cancer patients are especially at risk for surgery-related complications that lead to morbidity and mortality. Rockwood et al. define frailty as a multidimensional syndrome of diminished reserves that lead to increased vulnerability (3). A meta-analysis assessing the relationship between frailty and adverse outcomes across all surgical procedures found that frailty was associated with increased risk of surgical and perioperative complications, as well as readmission, postoperative discharge to skilled care, and mortality (4). Many of these adverse surgical outcomes have shown to be related to prolonged pain (5) and functional disability (6–9), as well as greater healthcare costs (10–12). Accordingly, identifying and mitigating frailty in cancer patients and other at-risk groups (*e.g.*, geriatric) are recommended to appropriately manage surgical risk (13, 14).

There are over 70 frailty assessments aimed at identifying or measuring the extent of frailty, many of which are multidimensional and include assessments of physical and cognitive function, nutritional status, comorbidities, and other factors that might affect the patient’s physiologic reserve or tolerance for surgery (13, 15). Clinicians’ impressions of frailty *via* bedside assessments have also demonstrated strong predictive capacity for identifying patients at risk of significant surgical morbidity or mortality (16). One strategy to manage surgical risk following identification of vulnerability is prehabilitation. Prehabilitation refers to assessments and interventions initiated prior to treatment to create physiologic and psychosocial buffers that can be protective against anticipated deconditioning, complications, and chronic morbidity that occur as a result of the treatment itself (17, 18). Contemporary prehabilitation is multimodal, often including a combination of exercise, enhanced nutrition, stress management, smoking cessation, and medical optimization strategies—strategies that are also commonly used to reduce frailty.

Systematic reviews and meta-analyses of prehabilitation prior to cancer surgery have reported encouraging findings, including improved physical fitness, length of stay, surgical complication rates, and HRQOL (19–24). In recent years, growing attention has been paid to patients who are frail, higher risk, and/or vulnerable to surgical complications, and thus likely to benefit most (25–27). For example, Barberan-Garcia et al. (26) conducted an RCT of prehabilitation in 174 ‘high-risk’ patients defined as older than 70 years and/or an American Society of Anesthesiologists score of III/IV, over half of whom were oncology patients. The intervention was feasible and safe, and prehabilitation reduced postoperative complications by half compared to the control group. Importantly, in a follow-up economic analysis, their intervention cost 389 Euro and yielded a six-fold reduction in risk of hospital readmissions at 30 days, collectively yielding a potential cost savings of up to approximately 800 Euro per patient (28). Aligned with these emerging data are implementation recommendations that include triaging strategies that prioritize prehabilitation for ‘at-risk’ or ‘frail’ patients for whom the benefits and cost effectiveness are likely to be greatest (29–31).

As evidence regarding the efficacy and potential healthcare savings for prehabilitation in cancer surgery continues to mount, consideration for clinical care pathways, delivery strategies, and required infrastructure and personnel are important pragmatic considerations for potential implementation. Data in these areas are lacking, spurring calls for pragmatic effectiveness trials of prehabilitation models of care (32). Pragmatic trials complement RCTs, the latter of which are considered the gold standard for assessing efficacy and causality, but whose methodological principles emphasize internal validity, often at the expense of generalizability to clinical practice. As such, public health and clinical research initiatives have increasingly sought to generate parallel ‘practice-based evidence’ to advise the development of intervention designs that can be applied in the real-world setting (33). Practice-based evidence can be derived from implementation science research methods, such as pragmatic trials, that assess intervention effectiveness in real-world settings and provide insight into the system’s capacity and preparatory needs for dissemination or scalability (34). The blending of experimental and implementation evidence has been suggested to target both internal and external validity and can offer important insight into implementation that cannot be well ascertained in conventional RCTs alone (34, 35).

To complement the growing RCT evidence, we designed a pragmatic trial of prehabilitation for people undergoing cancer surgery to advance the understanding of health professional engagement, delivery modality preference, and other insights related to the strategies, facilitators, and barriers of prehabilitation program implementation. Hereafter, we provide the trial protocol including adaptations related to the COVID-19 pandemic.

## Study Objectives

The primary objective of this study is to assess the feasibility of delivering a multimodal surgical prehabilitation service to surgical oncology patients. The secondary objectives are to explore the effectiveness of the program using clinical, physical, and patient-reported outcome measures. The specific research questions guiding this study design are listed in **Box 1**.

**BOX 1. Study Research Questions.**

Feasibility Research Questions:

RQ1a: How many referrals for prehabilitation will be received and what are the identified indications for prehabilitation?

RQ1b: Does a surgeon’s bedside assessment of frailty (as indicated by referral and reason for referral) correspond with established frailty indices?

RQ1c: What percentage of referred patients participate in prehabilitation?

RQ1d: What are the demographic and medical characteristics of patients who are referred to for prehabilitation?

RQ1e: What factors contribute to participants choosing either FBP or HBP?

RQ1f: What is the ‘prehabilitation window’ for participants (i.e., time from treatment decision to surgery)?

RQ1g: What is the adherence rate to the multimodal components defined by the prehabilitation protocols?

RQ1h: Is prehabilitation safe within a clinical model of care (i.e., number and nature of adverse events)?

RQ1i: What are the barriers and facilitators to prehabilitation?

RQ1j: What are the various costs and potential cost savings associated prehabilitation?

Exploratory Effectiveness Research Questions:

RQ2a: What changes in clinically relevant outcomes do participants experience by the week prior to surgery and up to 90 days after surgery?

RQ2b: Compared to usual care, what effect does prehabilitation have on peri- and postoperative outcomes (up to 90 days after surgery)?

(RQ, Research Question)

## Methods

### Design

**Figure 1:**
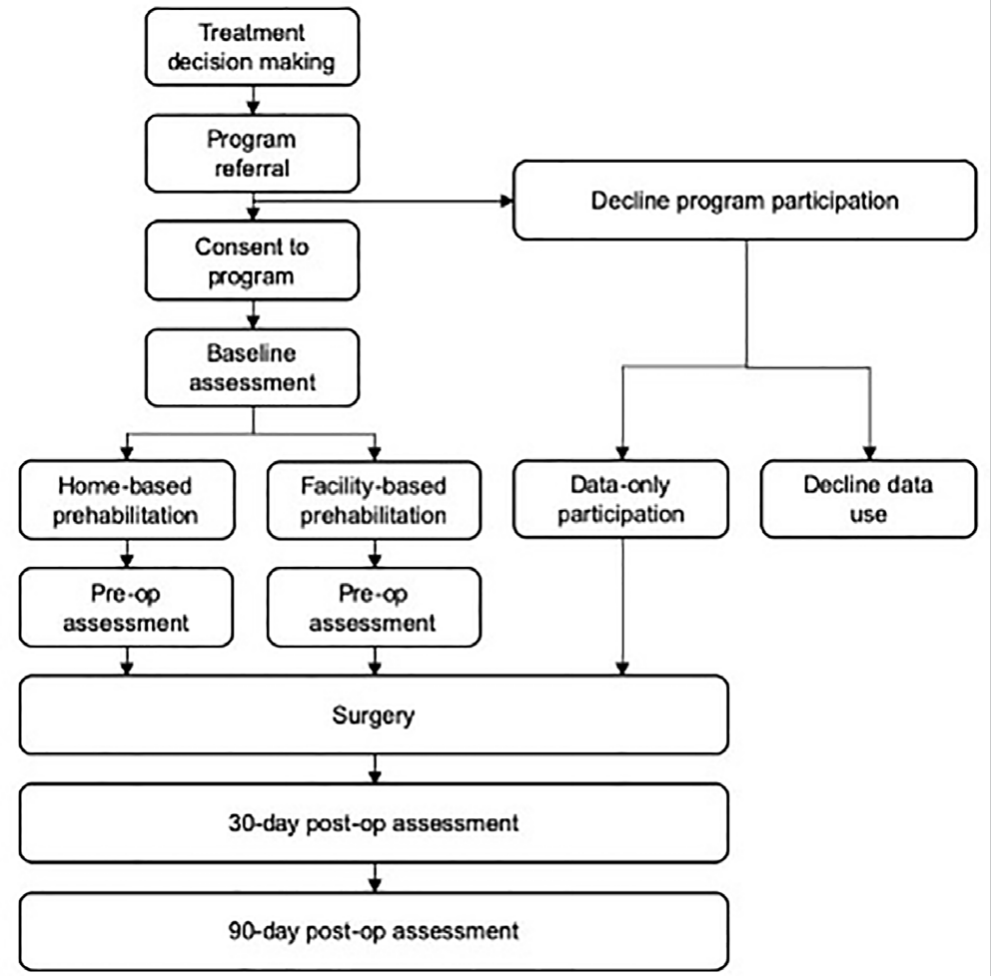
Participant flow.

This is a pragmatic, preference-based, non-blinded, non-randomized trial to assess the feasibility and estimates of effectiveness of a clinically integrated, multimodal prehabilitation program for frail surgical oncology patients in an urban academic health center in Toronto, Ontario, Canada. Participant flow throughout the study is presented in **Figure 1**. The initial study protocol and subsequent amendments related to the COVID-19 pandemic have been approved by the University Health Network research ethics board.

### Participants

Consistent with pragmatic trial methodology (34), broad inclusion criteria for study participation are employed for generalizability to the heterogeneity of patients that may be referred to a clinical service. Eligible patients for this study are: i) scheduled for cancer-related surgery; ii) 18 years of age or older; iii) fluent in English; and iv) referred by a health professional with an indication for prehabilitation (*e.g.*, higher-than-average risk candidate; marginal candidate for surgery due to perceived limited physiologic reserve; frail; deconditioned; **‘**other**’** with explanation).

### Sample Size

A period of trial enrolment, rather than target sample size, was selected to inform expected rates of referral for a clinical service. The trial anticipates receiving 150 referrals for prehabilitation over 12 months. We estimate that one third of all referred patients will decline the intervention but will consent to making their hospital records related to their pending cancer surgery available for research (hereafter referred to as ‘usual care’ participants).

### Outreach and Enrolment

A patient referral strategy for enrolment is adopted to model conventional clinical programming. To inform institutional stakeholders of the research project (*e.g.*, physicians and surgeons, physician assistants, nurses, and administrative assistants), a campaign of presentations, meetings, and emails pertaining to the study is conducted across surgical teams, in multidisciplinary rounds, and ambulatory clinics. Clinical teams receive information on the study**’**s objectives and methodology, including information on how to refer patients to the study, the referral form, and a prehabilitation program handout to review and distribute to patients. Clinicians are advised to introduce the study to patients whom they feel may be appropriate candidates for surgical prehabilitation at or near the time of treatment decision-making or during other medical visits associated with surgical planning (*e.g.*, comprehensive geriatric assessment). If the patient is interested in learning more or participating in the program, clinicians are advised to fax the study referral to the research team who subsequently contact the patient to discuss the study and obtain informed consent from agreeable and eligible patients (including usual care participants).

### Health History Interview and Baseline Assessment

At baseline, the research coordinator conducts a health history interview to ascertain information about their cancer diagnosis, planned surgery and related treatments (*e.g.*, neoadjuvant therapy), other injuries, illnesses and their associated treatments, previous experience with physical activity and exercise, nutrition and psychological stress. The health history interview aids in individualization of the prehabilitation programming and is supported by the following measures: the Charlson Comorbidity Index (CCI) (36); the Edmonton Frail Scale (EFS) (37); the Duke Activity Status Index (DASI) (38); the Canadian Nutrition Screening Tool (CNST) (39); the Perceived Stress Scale (PSS) (40), and the Godin Leisure-Time Exercise Questionnaire (GLTEQ) (41, 42). Finally, a 3-day food record is also used to quantify nutritional intake to aid dietary assessment and recommendations from the dietitian.

Peak aerobic fitness (VO_2peak_) is measured *via* a cardiopulmonary exercise test (CPET) using a cycle ergometer-based ramp protocol (43, 44) to determine safety and exercise parameters for participants engaging in high-intensity interval training (HIIT). Gas exchange is measured by indirect calorimetry *via* metabolic cart (TrueOne 2400, Parvo Medics, Sandy, UT, USA) and heart rate and rhythm are monitored continuously *via* 12-lead ECG (CASE, General Electric Healthcare, Chicago, IL, USA). Blood pressure, respiratory rate, and rating of perceived exertion are measured at the start of the test and routinely throughout.

### Prehabilitation Program

To accommodate individual factors that support program participation, prehabilitation is offered as either a facility-based or home-based intervention. Facility and home-based intervention delivery offer unique advantages and disadvantages that may relate to program participation and outcomes which are of particular interest to this study. In facility-based programming, health professional supervision can facilitate expedient adaptation and progression of the intervention to optimize patient safety and intervention efficacy (45). The disadvantages of facility-based programming relate to the accessibility of the facility (*e.g.*, distance, traffic, cost of fuel/parking, timing of facility-hours) and the general lack of program availability due to the institutional cost of intervention delivery (46, 47). Alternatively, home-based programs are less resource intensive for institutions to deliver and may impose fewer barriers to participant engagement which adds flexibility to accommodate schedules. A drawback of home-based programming is the absence of direct supervision which may limit intervention dose delivery, and consequently intervention efficacy, with the added reliance on potentially biased self-report measures to capture adherence and progress (48, 49).

In the present study, we sought to examine trends in delivery mode preference and participation and offered two streams of prehabilitation programming: home-based prehabilitation (HBP) and facility-based prehabilitation (FBP). To support patients in determining their preferred or optimal intervention setting, the research coordinator (who is also a health professional) engages in a shared decision-making conversation during the baseline assessment using the ‘choice, option, decision talk’ framework (50). Participants then continue with the baseline assessment oriented towards either HBP or FBP. Each intervention arm is similar in terms of intervention content (described further below) and primarily differs by the location of participation, where HBP participants engage with the intervention exclusively at home or their community and are remotely supported/counseled by telephone, whereas FBP participants engage in intervention *via* session occurring at the facility (*i.e.*, hospital) and at their home or community.

#### Exercise

Each participant’s exercise prescription is developed and delivered by a kinesiologist and individualized to the results and observations obtained during the baseline assessment. Participants in both groups receive a moderate intensity aerobic and resistance training prescription to be completed 3–5 times per week for 60 min per session. Exercises specific to the anticipated locoregional impairments associated with the pending surgery are also prescribed for FBP and HBP participants to be completed independently. Participants in FBP are encouraged to attend two facility-based sessions per week where the aerobic training includes HIIT using the 10 × 1 protocol (51), and on such days, resistance training using the facility’s equipment is commenced after a 10 min rest period. All home- or community-based exercise sessions are supported with the provision of a stability ball, resistance bands, and a manual free of charge, and are intended to be completed independently (*i.e.*, unsupervised). Prior to initiating the exercise program, all exercises are instructed and demonstrated in the prehabilitation program facility where participants have an opportunity to practice and receive feedback/corrections or alternate exercises. The kinesiologist communicates weekly with participants by telephone to support program compliance, record adherence, appropriate progression, and to address any barriers to exercise that may prevent participation. Details of the aerobic and resistance training programs, as well as the locoregional impairment-based exercises are provided in **Tables 1** and **2**, respectively.

**Table 1:** Exercise-based Total Body Prehabilitation.

	Home-based prehabilitation (HBP)	Facility-based prehabilitation (FBP)
Frequency	3–5 sessions per week (plus additional sessions for locoregional exercises as indicated)	
Intensity—Aerobic	MICT at 40–70% HRR or an RPE of 4–7/10	Home-based sessions are as per HBP. Facility-based sessions (twice weekly) employ the 10 × 1 HIIT protocol (10 cycles of exercise and recovery intervals, each interval is 1 min). During the first week, exercise intervals are 70–80% VO_2peak_ in session 1 and 75–85% VO_2peak_ in session 2 with recovery interval at a target an intensity of ≤50% VO_2peak_. In the second week and up to date of surgery, the exercise interval intensity target is set to 85–95% VO_2peak_.
Intensity—Resistance	2–3 sets of 8–12 repetitions at approximately 12–15 repetition maximum	
Time (duration)	Exercise sessions are intended to incorporate 25 min of aerobic and resistance training each plus a 5-min warm-up and cool-down. The total duration of training is intended to take approximately 60 min, but sessions may be divided into shorter bouts as needed.	
Type—Aerobic	The default modality of home-based training is brisk walking, or a low-intensity aerobic step class video developed and previously used by our team. Additional modalities of aerobic exercise may be used based upon the participants access (*e.g.*, attendance to a local fitness facility)	Home-based sessions as per HBP. Facility-based HIIT sessions use a treadmill or stationary cycle.
Type—Resistance	exercises targeting major muscle groups of the body (*e.g.*, shoulders, chest, upper/lower back, core, upper/lower legs).	
Progression—Aerobic	Progression of MICT will increase from the lower limit of the range (*e.g.*, 40% HRR) towards the upper limit of the range (*e.g.*, 70% HRR), and if required an increase in duration is implemented to progress the total aerobic training volume. Progression of HIIT is as per the familiarization and standard protocol described above, as well as progressing from the lower end of the standard range (85% VO_2peak_) to the upper limit (*e.g.*, 95% VO_2peak_)	
Progression—Resistance	Progression in resistance intensity occurs when 15 repetitions of a given exercise can be completed with only mild exertion.	

HIIT, High intensity interval training; HRR, heart rate reserve; MICT, moderate intensity continuous training; VO_2peak_, peak oxygen uptake (as determined during baseline cardiopulmonary exercise test).

**Table 2:** Locoregional/Targeted Preoperative Exercises.

Surgery	Description & Rationale	Training modalities
Abdo-thoracic (*e.g.*, lung resection, upper abdominal)	Exercises of the inspiratory muscles and diaphragm aim to increase the endurance, strength and performance of the inspiratory tissues. This has been shown to reduce pulmonary complications from abdo-thoracic surgery (52, 53).	Deep diaphragmatic breathing and inspiratory muscle training (IMT) with a threshold-loading device and nose plug (Threshold IMT, Respironics, Inc., Murrysville, PA, USA) Frequency: 5–7 times per week; Intensity: 4-7/10 on RPE scale. Duration: 15–30 minutes.
Urological (*e.g.*, prostatectomy, hysterectomy)	Training of the pubococcygeus, iliococcygeus, coccygeus, and puborectalis muscles, collectively referred to as the pelvic floor muscle exercise training has shown to reduce time to continence as well as the severity of incontinence postoperatively (19, 54).	Pelvic floor muscle exercises (contract and hold or 5–10 s) consistent with institutional strategies for radical prostatectomy rehabilitation Frequency: three sessions per day, every day; Intensity: maximal contraction for 10–20 repetition; Duration: ~5 min per session.
Breast (*e.g.*, lumpectomy, mastectomy)	Exercises targeting the upper quadrant and core are associated with early recovery of morbidity associated with resection and reconstruction (55).	Stretching and general strengthening of the shoulder, chest, and mid/upper back muscles consistent with institutional rehabilitation strategies for breast cancer surgery. Stretching is recommended daily and strength is incorporated into general conditioning protocol (described in **Table 1**).
Head and neck	Exercises of the pharyngeal muscles involved in speech and swallowing have primarily been used prior and during radiation and chemoradiation (56). Improvements in dysphagia and quality of life have been reported in patients undergoing treatment for head and neck cancer (57, 58).	Respiratory-swallowing coordination, postural exercises, tongue, jaw, and neck muscle strengthening (*e.g.*, supraglottic swallow, Masako technique). These comprise five swallowing exercises performed three sessions per day. Frequency: Daily; Intensity: not applicable; Duration: approximately 5 min per session

RPE, rating of perceived exertion.

#### Nutrition

A dietitian conducts an initial individualized nutrition assessment and counseling session within the first week of prehabilitation and again in the week prior to surgery. Each consultation is ~60 min and includes a review of the patient’s nutritional and weight history (including information from the 3-day diet record) and conversation regarding strategies to help the patient optimize or enhance the nutritional quality of the diet aligned with Canada’s Food Guide (59). Additionally, counseling regarding the maintenance of a healthy weight, minimizing excessive weight gain or weight loss, and addressing any nutrition-related questions or concerns specific to the pre and postoperative period is provided. To maintain protein sufficiency for exercise and prevent catabolism associated with the perioperative experience, participants are provided with 26 g packets of whey protein isolate, free of charge, to be consumed daily mixed in a beverage or food (ISOlution, Enhanced Medical Nutrition, Toronto, ON, CA) (60, 61). Participants are encouraged to contact the dietitian as needed for on-going support.

#### Stress Management and Behavioral Support

Within one week of initiating prehabilitation, a psychologist delivers a ~60-min psychoeducation session that focuses on stress management *via* relaxation, mindfulness, goal setting, and strategies to overcoming barriers to practice. In the week prior to surgery, participants are offered a second 60-min consultation with the psychologist to review their stress management experiences and provide further support for the acute perioperative period. To help participants with daily stress management practice, publicly available links to written and audio-based materials describing mindfulness, progressive muscle relaxation, deep breathing, and visualization are also provided.

#### Smoking Cessation

Participants that smoke are provided with information on the Canadian Cancer Society’s Smoker’s Helpline (www.smokershelpline.ca) for online programming and tools, as well as one-on-one counseling support. Smokers are also advised to speak to their local pharmacist and/or family doctor who can provide additional counseling, including education on the use of nicotine replacement therapy.

### Study Outcomes

Participant data to assess feasibility and derive estimates of effect are collected from the participants’ referrals, at the baseline assessment, within 1 week prior to surgery, and at 30 and 90 days postoperatively.

The total number of referrals and the rate at which they are received (per month) will be reported. To characterize the patients referred to the program, the following are collected from all referral forms (including usual care participants and those who decline research): referring surgical service; reason for referral; frailty level (*via* the Clinical Frailty Scale (3), embedded into the referral); cancer type; indicated surgery; referring healthcare practitioner type (*i.e.* physician, surgeon, clinic nurse, *etc.*), and participant demographics (age, sex, and general geographic location). The enrolment rate will be calculated as [# of enrolled participants]/[# of referred participants]. The frequency and reasons for declining participation in the study, declining prehabilitation (*i.e.*, usual care participants), and drop-out will be reported and compared using descriptive statistics of demographic and referral data.

Given the importance of scheduling and timing for prehabilitation relative to the date of surgery, several relevant time periods will be reported. The time from program referral to the date of surgery will be reported and is referred to as the ‘prehabilitation window’. We will also report the total preoperative period (time between consent for surgery and date of surgery) and prehabilitation duration (time from baseline assessment to surgery).

Reporting of the exercise prescription parameters and adherence to the programming will follow the Consensus on Exercise Reporting Template (62). Adherence to home and facility-based exercise sessions are recorded *via* attendance and standardized logbooks capturing training activity completed by the research coordinator. Adherence to stress management, nutrition plan, protein consumption, and utilization of smoking cessation tools (as required) is recorded weekly using a logbook within the participant manuals. Healthy eating practices advised by the dietitian are also assessed by a 3-day diet record in the week prior to surgery. Safety or adverse events related to prehabilitation are discussed during weekly communication between the participants and the research coordinator. Reporting and grading of adverse events will follow the Common Terminology Criteria for Adverse Events version 5.0 (63).

Estimates of program effectiveness are derived from a combination of patient-reported and functional performance measures, as well as clinical information from the medical record at each of the study timepoints. Aerobic functional capacity is measured using the Six-Minute Walk Test (6MWT) (64) and musculoskeletal functional capacity is assessed *via* grip strength using an isometric dynamometry (Jamar, Sammons Preston, Bolingbrook, IL, U.S.A.) according to established protocols (44). Body mass (kilograms) and height (meters) are measured using standardized procedures and are used to calculate body mass index (BMI, kg/m^2^). Body fat percentage, fat and fat free mass, impedance, resistance, and phase angle are recorded are measured *via* bioelectrical impedance analysis (mBCA 514, Seca, Hamburg, Germany) (65). HRQOL is measured using the 12-item Short Form Health Survey (SF-12) (66, 67) and the EuroQol 5 Dimensions 5 Levels (EQ5D-5L) (68) The Patient Health Questionnaire (PHQ-9) is used to assess depression (69). The EFS, PSS, and GLTEQ are also re-administered at each follow-up timepoint. Postoperative length of stay in number of days (including any readmissions) is recorded from the medical record. Complications, including mortality, are reported according to the Clavien-Dindo classification (70). All health events that require readmission will also be documented. Complication and health event data are extracted from the medical record at the 30^th^ and 90^th^ postoperative day for each participant.

Economic evaluation will be conducted from the perspectives of the individual and the hospital. Cost for an individual prehabilitation participant will be calculated on two fundamental components: the quantity of resources consumed and the unit cost of those resources related to prehabilitation. The EQ5D-5L will be used for the cost–effectiveness analysis as the health effect to determine the quality-adjusted life years (QALY) for the 90-day follow-up time period. The calculated participant costing and QALY will be used to determine the incremental cost–effective ratio (ICER). ICER will be calculated as a ratio of the difference in patient costing and the difference in QALY between FBP and HBP [ICER = (cost_FBP_ – cost_HBP_)_/_(QALY_FBP_ – QALY_HBP_)]. This will be calculated for both the program (measured cost for delivery of FBP and HBP) and patient perspective. Patient-perspective costing is measured by a patient-reported cost-diary that includes: direct healthcare cost (impact of the interventions on the use of healthcare services, such as visits to the general practice, specialist care, prescribed medication); direct non-healthcare costs (cost incurred by the patient and the family, such as cost of over-the-counter medication, cost of health activities, hours of paid and unpaid household help, transportation, and value of other out-of-pocket expenses, with specifics on exercise-related expenses); and indirect costs (value of productivity lost due to illness-related absence, including number of days absent from work, days lost from housekeeping, and other daily activities). A cost–impact from the perspective of the hospital will be conducted based on surgery-related hospital length of stay, readmission frequency, and length of stay of readmission(s) will be used to determine cost differences between those that participate in prehabilitation versus usual care participants. Cost impact will be estimated by applying the unit cost of an inpatient hospital day to the differences for participants that enrolled in prehabilitation and those that did not. Data from the Canadian Institute for Health Information on average cost of hospital stay will be used for the respective year.

In the second year of the study, prehabilitation participants will be asked to participate in semi-structured interviews conducted by telephone or in-person. The purpose of the semi-structured interviews is to capture insights about participant satisfaction, as well as the facilitators and barriers to prehabilitation engagement. To reach saturation for identifying meta-themes within a heterogenous population, a purposive sample of at least 15 participants per study arm will be sought to identify prevalent and salient themes related to study experiences. Qualitative content analysis will be conducted to identify barriers and facilitators for prehabilitation participation and engagement will be conducted using semi-structured interviews.

### Analytic Plan

The analytic plan is described for prehabilitation implementation feasibility outcomes and exploratory analyses of prehabilitation effects. In line with comparative effectiveness research, presentation of confidence intervals will be emphasized for the purpose of accurately reflecting the actual data as well as directly addressing the uncertainty of the data. All quantitative analyses will be conducted in R (R Foundation for Statistical Computing, Vienna, Austria) and an alpha of.05 will be used.

Demographic and disease characteristics of all referred patients, as well as prehabilitation and usual care participants**’** will be summarized with appropriate parametric and non-parametric statistics. Reasons for ineligibility, declined participation in the study or intervention, as well as reasons for choosing FBP or HBP will be tallied. Group comparisons for referral information (surgical service, type of cancer, type of surgery, age, sex, and geographic location) will be assessed by one-way analysis of variance (ANOVA) for continuous and Chi-square test for categorical variables and described across FBP, HBP, usual care, and participants who decline participation. Baseline demographic and disease-related variables will be compared between study participants (FBP, HBP and usual care) ***via*** one-way ANOVA for continuous variables and Chi-square test for categorical variables.

Adherence to the interventions will be summarized dichotomously as meeting or not meeting the prescribed intervention components across each domain (exercise, nutrition, psychology, and smoking cessation). Reasons for deviations will be thematically categorized and summarized by frequency and percentage. Retention rates will be calculated as a total percentage of dropouts at the presurgical time point to the total participants enrolled for FBP and HBP. Reasons for dropout will be summarized using frequencies and percentage for each prehabilitation arm. Reported safety or adverse events will be summarized using frequencies and percentage for each group.

To provide an estimate of effect of HBP and FBP, point estimates and 95% confidence intervals will be calculated for changes in physical fitness, patient-reported outcomes from baseline to the 90-day time point using linear mixed effect models. Estimated mean hospital length of stay for HBP, FBP, and usual care, as well as between-group differences, will also be conducted using a linear mixed effect model. Incidence rate ratios and estimating rate differences for postoperative complication, readmission, and morbidity for prehabilitation in reference to usual care at 30 and 90 days after surgery will be made using Poisson regression. Tukey HSD will be used to adjust for multiple comparisons. In the presence of outliers, bootstrapping regression coefficient methodology will be done to obtain valid confidence intervals.

Semi-structured interviews regarding participant satisfaction as well as facilitators and barriers with the intervention will be transcribed verbatim and undergo qualitative content analysis. Initial transcript sample readings will be independently done by two researchers. Preliminary themes will be noted, and differences will be resolved, and duplications will be eliminated. Themes and content will be analyzed descriptively. Coding, linking, and retrieving the qualitative data will be conducted using NVivo software (QSR International, Melbourne, AUS).

### Protocol Adaptations in Response to the COVID-19 Pandemic

COVID-19 containment measures have reduced elective surgery volumes around the world. Reduced surgical capacity has led to longer wait times for elective procedures and patients are experiencing declining physiological and psychosocial health in the unsettling context of social distancing, community service closures, and economic hardship. This loss in health is likely to be most profound for older patients and those with complex medical needs. Consequently, the extended waiting time is likely to negatively impact disease progression and surgical tolerance that may lead to higher rates of adverse surgical outcomes, ultimately compounding COVID-19-related health system strain. Given that prehabilitation may play an important role in mitigating the deterioration of health and well-being during extended surgical wait times, this study implemented several amendments to accommodate pandemic-related restrictions and barriers to healthcare in an attempt to maintain the opportunity for prehabilitation participation for planned but unscheduled, or delayed, cancer surgery. A summary of the amendments approved by the institutional ethics board is provided in **Box 2**.

**BOX 2. COVID-19 Pandemic-Related Study Accommodations.**

1. Extension of enrolment period by at least 6 months to accommodate pauses in research and to initiate contactless study protocols

2. Accept form-fillable PDF referrals by email from clinicians (*versus* referrals by fax)

3. Informed consent is obtained verbally, by phone, with informed consent documentation emailed to participants to be completed and returned at their next hospital visit (*e.g.*, date of surgery, post-operative clinic visit)

4. All interactions between participants and study staff, including the baseline assessment, are completed by telephone or web conferencing (Microsoft Teams, Redmond, Washington, USA)

5. Exercise equipment, manuals, and protein supplementation are mailed to participants

6. All exercise sessions are intended to be conducted at-home and employ the same exercise parameters for HBP described in **Table 1**. Additional emphasis on strategies to maintain social distancing is provided for those who are engaging in outdoor exercise.

7. Study outcomes requiring an in-person assessment (*e.g.*, 6MWT, body composition, grip strength) are omitted during in-person research restrictions, and only data derived from questionnaires and the electronic medical record are collected for exploratory analyses of effectiveness

## Discussion

As the evidence supporting prehabilitation for cancer surgery grows, questions about if and how it may be integrated into standard of care have followed. This protocol describes a study aimed at advancing implementation evidence to complement ongoing RCTs that target efficacy outcomes. Collectively, these will inform clinicians and researchers about the value and feasibility of clinically integrated prehabilitation for people with cancer. Importantly, to maximize generalizability to clinical care, as well a sustainable model of delivery, this study uses a referral-based enrolment strategy for a broad range of oncology patients who are identified as frail or vulnerable to adverse surgical outcomes. Related to our objectives of determining the appropriateness of referrals, a key learning outcome of our research will be the estimated frailty of referred patients using the Clinical Frailty Scale (3) and how those ratings correspond with other markers of frailty and performance, as well as prehabilitation adherence and study retention.

Within the context of a pragmatic trial design, we elected to offer two streams of prehabilitation, FBP and HBP, which are selected by participants using a shared decision-making strategy with a member of the research team. Advantages to the preference-based design include better motivation and compliance with an intervention, and subsequently more favourable experiences and outcomes than they may have in their non-preferred study arm (71). Moreover, preference for a study arm can enhance external validity and generalizability to clinical practice (72–75). In clinical settings, patients’ preferences, facilitators, and barriers to participation, intervention efficacy, and equitable access to services are fundamental considerations in designing and delivering health services and are core outcomes for implementation research (76–78). As such, offering both FBP and HBP options are likely to satisfy patients’ needs and capacities to ensure greatest benefit to all who are referred and examination of participation across study arms will yield novel and important insight into delivery models.

The COVID-19 pandemic has created unprecedented, systemic delays in surgical procedures that are negatively affecting elective surgery patients worldwide. Evidence is rapidly mounting regarding the significant physiological and psychosocial stress due to progressive symptoms and disease status, physical inactivity, poor nutrition and economic hardship for patients awaiting surgery. These, unfortunately, are compounded with uncertainty of surgical outcomes, social isolation, fear of COVID-19 infection, and lack of access to healthcare supports that collectively will likely contribute to a substantially higher risk of surgical complications, longer and poorer recovery, and greater health system cost. Strategies to mitigate rapidly declining preoperative health are needed, especially to manage the eventual surge in surgical demand as postponed procedures are resumed or become urgently required. Prehabilitation represents an important strategy to combat the pandemic-related patient and health system challenges of surgical delays given its capacity to adapt to a contactless model of care as well as providing ongoing support to those with distance-related barriers or apprehension about visiting facilities (79, 80).

There are several strengths of this study. First, the pragmatic, preference-based trial design with robust implementation feasibility outcomes and measures of effectiveness will add important information to the prehabilitation literature that is currently lacking in these areas. Second, the prehabilitation interventions are multimodal and comprehensive within each modality intended to replicate gold-standard practice. Moreover, the interventionists represent the appropriate scopes of practice and clinical professions most qualified and likely to be involved in an interprofessional, multimodal clinical prehabilitation service. Third, by including a usual care arm, we have a control comparator for effect size estimates. Fourth, we have amended our research protocol to respond to the evolving context of the COVID-19 pandemic by pivoting towards contactless study participation. This study also has noteworthy limitations. The sample size will likely lack the statistical power to draw precise conclusions about the effect of the interventions. Similarly, in the absence of an RCT design, our interpretations of comparisons with usual care participants may be limited due to group differences in those who do versus those who do not wish to engage in prehabilitation. Interpretation of the findings will also be limited to the types of surgeries for which prehabilitation precedes which may be skewed to the physicians and healthcare teams who are in favour of prehabilitation and refer patients to our study. This highlights potential sampling and participation biases as participants will more likely be referred and participate in our program if their healthcare team implicitly endorses it by virtue of discussing it and making a referral. Similarly, the breadth of cancer surgeries and their extreme heterogeneity within a relatively small sample will limit sub-group analyses related to estimates of intervention effect.

## Conclusion

Prehabilitation has become an intriguing health intervention for people undergoing cancer surgery with growing evidence of its efficacy, especially in frail and at-risk populations. Despite growing interest in implementation, few studies have evaluated the feasibility of implementation and characteristics of models of care that resemble an integrated clinical service. The present study will contribute important implementation evidence regarding surgical prehabilitation programming while providing estimates of effect for two intervention models in frail and at-risk people with cancer.

## Data Availability Statement

The original contributions presented in the study are included in the article/supplementary material. Further inquiries can be directed to the corresponding authors.

## Ethics Statement

The studies involving human participants were reviewed and approved by the University Health Network. The patients/participants provided their written informed consent to participate in this study.

## Author Contributions

All authors listed have made a substantial, direct and intellectual contribution to the work, and approved it for publication.

## Funding

This study was funded by University Health Network Academic Medical Organization (AMO) Innovation Fund.

## Conflict of Interest

The authors declare that the research was conducted in the absence of any commercial or financial relationships that could be construed as a potential conflict of interest.
